# Ecological Adaptation Mechanisms Underlying Successful Plant Reproduction

**DOI:** 10.1002/advs.202520550

**Published:** 2026-04-16

**Authors:** Hang Zhao, Shenghui Huo, Yue Xing, Shuxin Zhang, Guilin Li, Yihui Liu, Chongyang Jin, Fuguang Li, Xiaoyang Ge

**Affiliations:** ^1^ School of Life Sciences Shandong Key Laboratory of Wetland Ecology and Biodiversity Conservation in the Lower Yellow River Qufu Normal University Qufu China; ^2^ State Key Laboratory of Cotton Bio‐Breeding and Integrated Utilization Institute of Cotton Research of Chinese Academy of Agricultural Sciences Anyang China

**Keywords:** ecological adaptation, flowering, plant reproduction, receptor‐like kinases, small peptides

## Abstract

As sessile organisms, plants have evolved sophisticated adaptive strategies to withstand fluctuating and often unpredictable environments. These strategies optimize reproductive traits, enabling plants to sustain reproduction under adverse conditions. Crucially, this environmentally driven reproductive plasticity not only ensures species survival but also offers avenues to enhance crop yield and quality. Addressing a critical gap in understanding how reproductive physiology integrates environmental adaptation, this review synthesizes recent advances on how external signals (e.g., light, temperature) interact with endogenous regulators (e.g., phytohormones, transcription factors, small peptides, receptor‐like kinases) to modulate plant reproductive processes. It encompasses the full spectrum of reproductive biology, spanning floral transition, floral organ specification, gametogenesis, and fertilization. Furthermore, we discuss the potential applications of reproductive biology in future crop breeding and outline key research directions to advance the field.

## Graphical abstract

During floral induction, various environmental and endogenous signals converge to regulate the florigen protein, which is transported from leaves to the SAM to initiate flowering. Within the SAM, a complex network of receptor kinases and small peptides orchestrates floral development with high spatiotemporal precision. These regulatory mechanisms govern critical processes, including floral meristem maintenance, organ development, and gamete fusion.

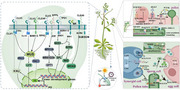

## 1 Introduction

Plant reproductive physiology underpins reproductive success and is a primary determinant of crop yield and productivity. It encompasses floral induction, flower initiation, and the development of male and female gametophytes. Over millennia of evolution and domestication, plants have acquired the capacity to perceive subtle environmental fluctuations—particularly in light and temperature—and to integrate them through spatiotemporally precise regulatory networks. This integration enables the execution of reproductive processes at developmentally optimal stages, thereby enhancing adaptability and ensuring reproductive success.

Flowering represents the central switch from vegetative to reproductive growth in angiosperms. It is governed by environmental inputs (e.g., photoperiod, temperature, circadian rhythms) and intrinsic cues (e.g., developmental age, endogenous hormones, sugars). These signals converge on the central integrator *FLOWERING LOCUS T* (*FT*), which transmits the floral stimulus to the shoot apical meristem (SAM) via the vascular system. Upon arrival, FT engages with 14‐3‐3 scaffold proteins to assemble the florigen activation complex (FAC) alongside the transcription factor FD [1]. This nuclear complex drives the transcriptional activation of meristem identity genes, such as *LEAFY (LFY*) and *APETALA1* (*AP1*), thereby triggering the floral transition [2, 3, 4, 5, 6, 7]. Subsequent reproductive processes—such as floral meristem maintenance, organ development, and gametophyte fusion—are coordinated by regulators of the ABC model (not revisited here) and by diverse signaling peptides and their cognate receptor kinases.

Despite extensive progress, a systematic framework explaining how plant reproductive physiology adapts to environmental signals remains lacking. This review addresses that gap by elucidating molecular mechanisms through which plants perceive and integrate temperature and light cues to finely tune flowering—a key adaptive strategy under climate change. We also examine the emerging role of peptide–receptor kinase modules in reproductive phase transitions. By synthesizing these regulatory layers, we propose an integrated framework of the signaling networks that confer flowering plasticity in a warming world. This synthesis deepens fundamental understanding of adaptive reproduction while providing a theoretical foundation for molecular breeding strategies aimed at optimizing flowering time to improve crop resilience and productivity.

## 2 Ambient Temperature‐Mediated Flowering Plasticity

### 2.1 Floral Integrators

**FIGURE 1:**
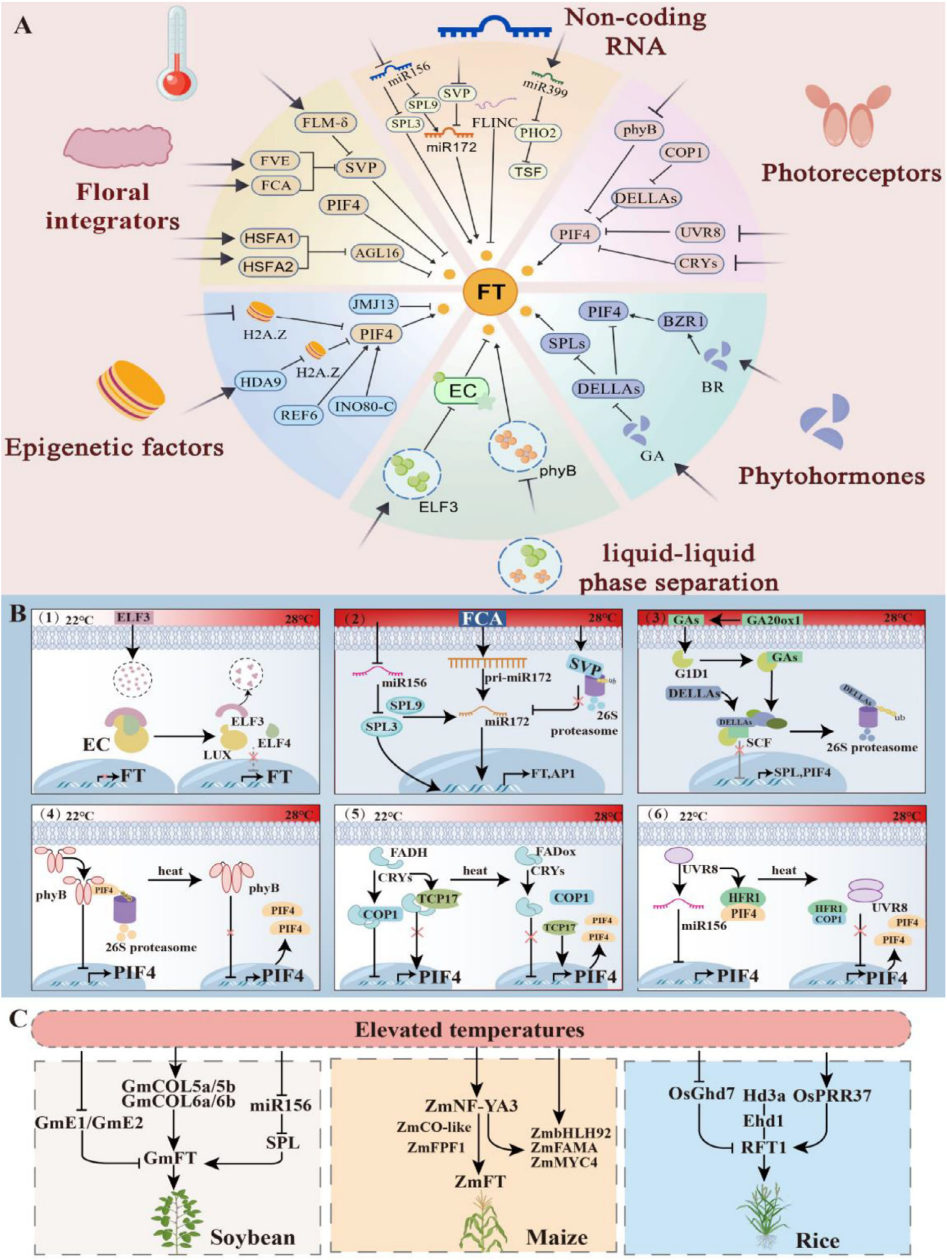
Mechanisms of temperature‐mediated flowering in plants. (A) Temperature‐mediated flowering plasticity is governed by six distinct regulatory pathways, including floral integrators, epigenetic modifiers, liquid–liquid phase separation, phytohormones, and non‐coding RNAs. (B) Flowering time is dynamically regulated by the Evening Complex (EC), non‐coding RNAs, GA signaling, and light‐responsive regulators through temperature‐activated signaling cascades. (C) Mechanistic basis of temperature‐dependent flowering in major crop species, including soybean, maize, and rice.

Over the past decade, *Arabidopsis thaliana* has provided extensive insights into the regulation of flowering by ambient temperature. As a central integrator within this complex regulatory network, FT converges multiple pathways to mediate thermosensory responses. Elevated temperatures stabilize transcriptional regulators such as the bHLH factor PHYTOCHROME INTERACTING FACTOR 4 (PIF4) and autonomous pathway components FCA (FLOWERING LOCUS CA) and FVE (FLOWERING LOCUS VE). Accumulated PIF4 binds directly to the *FT* promoter, activating its transcription and accelerating flowering [5]. Meanwhile, FCA and FVE repress SHORT VEGETATIVE PHASE (SVP), thereby relieving SVP‐mediated repression of *FT* and promoting floral transition [8]. Importantly, SVP itself undergoes temperature‐dependent degradation. At high temperatures, it is ubiquitinated by a CUL3‐based E3 ligase complex (CRL3) [9, 10], leading to loss of repression on FT and promoting thermally induced flowering [11]. Collectively, these findings reveal a multilayered regulatory framework for thermosensory flowering, rather than isolated effects of individual genes (Figure 1A,B and Table 1).

**TABLE 1:** Functional roles of flowering‐related factors in plants.

Gene name	Molecular function	Biological function	Ref.
*FT* (*FLOWERING LOCUS T*)	Induce *LFY* and *AP1* expression	Early flowering	[2, 3, 4, 5]
*SOC1* (*SUPPRESSOR OF OVEREXPRESSION OF CONSTANS 1*)	Induce *LFY* and *AP1* expression	Early flowering	[2, 3, 4, 5]
*LFY* (*LEAFY*)	unknown	Early flowering	[2, 3, 4, 5]
*AP1* (*APETALA1*)	unknown	Early flowering	[2, 3, 4, 5]
*PIF4* (*PHYTOCHROME INTERACTING FACTOR 4*)	Combining with the *FT* promoter to activate the transcription of *FT*	Early flowering	[5]
*FCA* (*FLOWERING LOCUS CA*)	Represses *Short Vegetative Phase* (*SVP*) expression	Early flowering	[8]
*FVE* (*FLOWERING LOCUS VE*)	Represses *SVP* expression	Early flowering	[8]
*SVP*	Represses the transcription of *FT*	Late flowering	[8]
*FLM‐β*	Form a complex with *SVP*	Late flowering	[9, 13, 14]
*FLM‐δ*	Ubiquitinate and degrade *SVP*	Early flowering	[9, 13, 14]
*AGL16* (*AGAMOUS LIKE 16*)	Negative regulation of *FT*	Late flowering	[15]
*HSFA1*	Represses *AGL16* transcription	Early flowering	[15]
*HTA8*, *HTA9*, and *HTA11*	Encodes histone variant *H2A.Z*	Late flowering	[16]
*COMPASS*‐like	Combine to *PIF4*	Early flowering	[22]
*AtGA20ox1* and *AtGA3ox1*	Accumulate active *GA4*	Early flowering	[33, 34]
*GID1* (*Gibberellin Insensitive Dwarf 1*)	Combine to activate *GA4* and specifically recognizes and recruits the *SCFSLY1*/*GID2* complex	Early flowering	[35, 36, 37]
*COP1*	Mediated ubiquitination pathway	Early flowering	[38, 39]
*MED15*	Degradation of *DELLA* protein	Early flowering	[38, 39]
*BZR1*	Promotes *BR* signal transduction	Early flowering	[40]
*BIN2*	Represses *BR* signal transduction	Early flowering	[41, 42]
*DWF4*	Unknown	Late flowering	[41, 42]
*CO* (*CONSTANS*)	Unknown	Early flowering	[46]
*TCP17*	Promote *PIF4* transcription	Late flowering	[50]
*UVR8*	Represses the activity of *PRC2* complex and stabilizes the *bHLH* protein *HFR1*	Late flowering	[54]
*H3K27me3*	Promotes *miR156* expression	Late flowering	[54]
*ELF3*	Combine *ELF4* and *LUX* to form *EC*	Late flowering	[57]
*ELF4*	Combine *ELF3* and *LUX* to form *EC*	Late flowering	[57]
*LUX*	Represses *PIF4* and *PIF5* transcription	Late flowering	[57]
*CCA1* (*Circadian Clock Associated 1*)	Promotes *PIF4* expression	Early flowering	[73]
*LHY* (*Late Elongated Hypocotyl*)	Promotes *PIF4* expression	Early flowering	[73]
*SHB1* (*SHORT HYPOCOTYL UNDER BLUE1*)	Interacts with *CCA1* and *LHY*	Early flowering	[73]
*GA20ox1*, *GA3ox1*	Promote the accumulation of active gibberellin *GA4*	Early flowering	[76]
*AtGA20ox2*	Promote *GA* expression	Early flowering	[76]
*E1*, *E2*	Unknown	Late flowering	[67]
*GmFT2a*, *GmFT5a*	Unknown	Early flowering	[67]
*GmCOL5a*/*5b*, *GmCOL6a*/*6b*	Unknown	Early flowering	[67]
*Ehd1* (*Early Heading Date 1*)	Activate *Hd3a*/*RFT1* transcription	Early flowering	[68]
*Hd3a*/*RFT1*	unknown	Early flowering	[68]
*OsGhd7*	unknown	Late flowering	[71]
*OsPRR37*	unknown	Early flowering	[72]
*ZmNF‐YA3*, *ZmFT‐like12*, *ZmFPF1*	Combine to form a complex	Early flowering	[69]

MADS‐box transcription factors are also central to temperature‐regulated flowering. A key regulator, FLOWERING LOCUS M (FLM), undergoes temperature‐dependent alternative splicing to produce antagonistic isoforms FLM‐β and FLM‐δ. At low temperatures, splicing factor SF1 promotes FLM‐β accumulation while suppressing FLM‐δ formation [12]. The predominant FLM‐β isoform partners with SVP to repress flowering. By contrast, elevated temperatures induce an isoform switch favoring FLM‐δ, disrupting the SVP–FLM‐β complex and promoting SVP degradation via ubiquitination [9, 13, 14]. This shift relieves FT repression, thereby enabling thermosensitive flowering. This splicing‐based mechanism is evolutionarily conserved, allowing plants to synchronize reproduction with seasonal thermal conditions—avoiding frost damage at low temperatures while accelerating flowering under warmer conditions to secure reproductive success. Another MADS‐box factor, AGL16, acts as a negative regulator of *FT* and flowering. Elevated temperatures activate HSFA1‐family and HSFA2 transcription factors, which repress AGL16 expression, thereby facilitating floral induction [15]. Collectively, these results demonstrate that temperature‐dependent flowering is orchestrated through a complex, adaptive regulatory system in which FT serves as the central hub. This system enables plants to accurately sense and respond to thermal signals, ensuring that flowering occurs at the most favorable developmental stage.

### 2.2 Epigenetic Factors

Histone modifications constitute a central epigenetic mechanism regulating high‐temperature‐induced flowering, primarily by modulating *FT* expression [16, 17]. A key regulator in this process is JMJ13, a Jumonji C (JmjC) domain‐containing H3K27me3 demethylase. Loss‐of‐function *jmj13* mutants exhibit accelerated flowering under elevated temperatures, accompanied by *FT* upregulation, suggesting that JMJ13 fine‐tunes *FT* expression as a temperature‐responsive regulator [17]. Similarly, the histone variant H2A.Z, encoded by *HTA8*, *HTA9*, and *HTA11* in *Arabidopsis*, plays a crucial role in thermal regulation of flowering [16]. Under normal conditions, H2A.Z incorporation into nucleosomes blocks transcription factor binding at the *FT* promoter, thereby repressing *FT* transcription and delaying flowering. Elevated temperatures trigger H2A.Z eviction from chromatin, releasing this repression and promoting flowering [16].

Beyond *FT* regulation, histone‐modifying enzymes mediate thermal responses through two primary mechanisms: (i) controlling *PIF4* transcription and (ii) modulating *PIF4* binding to its downstream targets. For instance, temperature‐dependent nuclear translocation of HISTONE DEACETYLASE 9 (HDA9) enables its interaction with POWERDRESS (PWR), forming a complex that deacetylates histone H3 at the *PIF4* promoter. This remodeling enhances *PIF4* transcription, promoting flowering under high temperatures [18, 19, 20, 21]. In parallel, histone‐modifying enzymes dynamically adjust the binding affinity of *PIF4* to its targets in response to temperature [22, 23]. *PIF4* recruits the INO80 chromatin remodeling complex (INO80‐C) to flowering‐related loci, where it collaborates with COMPASS‐like histone methyltransferase complexes to activate transcription and induce floral transition [22]. Likewise, elevated temperatures increase the expression of the histone demethylase REF6, which interacts with *PIF4* to enhance its binding to flowering regulators [23].

Although substantial progress has been made in elucidating histone‐mediated epigenetic control of thermoresponsive flowering, major gaps remain in identifying the full repertoire of regulatory proteins and downstream targets. The integration of 3D genomics and single‐cell multi‐omics will be essential to decipher spatiotemporal chromatin architecture in thermal responses. Cutting‐edge tools such as high‐resolution chromatin conformation capture, single‐cell epigenomic profiling, and live‐cell imaging promise to reveal how temperature cues reprogram chromatin states to fine‐tune flowering time. Such insights will not only advance mechanistic understanding of epigenetic regulation but also identify novel molecular targets for crop breeding aimed at climate adaptation.

### 2.3 Non‐Coding RNAs

Multi‐omics analyses under varying thermal regimes have identified small RNAs (sRNAs) as pivotal mediators of temperature‐responsive flowering. Among these, microRNAs (miRNAs) and long non‐coding RNAs (lncRNAs) represent two major classes of regulators [24, 25, 26]. miR156 is a master regulator of the vegetative‐to‐reproductive phase transition, acting through suppression of *SQUAMOSA PROMOTER BINDING PROTEIN‐LIKE (SPL)* genes [27, 28, 29, 30]. Elevated temperatures reduce *miR156* expression, thereby releasing its repression of the *SPL–FT* pathway and promoting floral transition [25]. Evolutionary conservation of this mechanism is illustrated by *miR535*, a banana homolog of *miR156*, which similarly modulates *SPL* expression under thermal stress [31]. By contrast, miR172 is strongly induced by high temperature through multiple pathways, including temperature‐dependent accumulation of the RNA‐binding protein FCA and the transcription factor SPL9, which together activate *miR172* transcription. This circuit is further reinforced by heat‐triggered degradation of the floral repressor *SVP*, which normally inhibits *miR172* expression, thereby establishing a robust positive feedback loop that accelerates flowering. In parallel, miR399 promotes flowering under elevated temperatures through the *miR399–PHO2* module, which upregulates *TWIN SISTER OF FT* (*TSF*), a functional homolog of *FT*, providing an additional pathway for thermoresponsive control. Collectively, these findings highlight an intricate miRNA network that ensures precise flowering regulation under fluctuating temperatures (Figure 1A).

In addition to miRNAs, lncRNAs (>200 nt) have recently emerged as key regulators of temperature‐mediated flowering [32]. Large‐scale transcriptome profiling has identified approximately 50 heat‐responsive lncRNAs, including natural antisense transcripts (NATs), intronic lncRNAs, and long intergenic non‐coding RNAs (lincRNAs). Among them, the flowering‐associated lincRNA FLINC (AtLnc428) is particularly notable [24]. Loss‐of‐function mutants *flinc* display early flowering and reduced temperature sensitivity, accompanied by increased *FT* expression, indicating that FLINC normally represses *FT* under fluctuating thermal conditions [24]. Although these findings establish lncRNAs as novel components of the thermosensory network, how FLINC and other temperature‐responsive lncRNAs perceive and transmit thermal signals to the flowering cascade remains poorly understood, highlighting an important avenue for future research.

### 2.4 Phytohormones

Elevated ambient temperatures also regulate flowering time through dynamic modulation of endogenous hormone levels and associated signaling cascades. A central mechanism involves the thermal induction of gibberellin (GA) biosynthesis, where high temperatures strongly upregulate *AtGA20ox1* and *AtGA3ox1*, two rate‐limiting enzymes in GA_4_ production [33, 34]. Bioactive GA_4_ binds to the GID1 (GIBBERELLIN INSENSITIVE DWARF1) receptor, triggering the assembly of the SCF^SLY1/GID2^ E3 ubiquitin ligase complex. This complex targets DELLA proteins—core repressors of GA signaling—for proteasomal degradation, thereby derepressing flowering activators such as *miR172*, *SPL* transcription factors, and *PIF4* [35, 36, 37]. Intriguingly, high temperatures can also promote DELLA degradation independently of GA. For instance, COP1‐mediated ubiquitination and the transcriptional coactivator MED15 both facilitate DELLA destabilization under elevated temperatures [38, 39]. Thus, DELLA proteins function as integrative hubs that converge multiple hormonal and environmental signals to orchestrate flowering responses to thermal stress (Figure 1A,B).

In addition to gibberellins, brassinosteroids (BRs) are pivotal in high‐temperature‐induced flowering through regulation of *PIF4* expression and protein stability. Elevated temperatures enhance the nuclear accumulation of BZR1, a positive regulator of BR signaling, which increases *PIF4* transcription and promotes floral induction [40]. Concurrently, BIN2, a negative BR regulator, mediates the nuclear localization of the heat shock transcription factor HSFA1d, which interferes with the phyB–PIF4 interaction and stabilizes *PIF4*. Stabilized *PIF4* then competitively displaces BES1 from BR‐responsive elements (BRREs) in the *DWF4* promoter, suppressing this floral repressor and facilitating the transition to flowering [41, 42](Figure 1A,B and Table 2).

**TABLE 2:** Protein interactors of early maturity factors in plants.

Protein interaction	Molecular function	Ref.
SVP‐CRL3	Promotes *FT* expression	[9, 10]
SVP‐FLM‐δ	Promotes the ubiquitination and degradation of SVP	[9, 13, 14]
HDA9‐PWR	Promotes *PIF4* expression	[18, 19, 20, 21]
PIF4‐INO80	Promotes the binding of PIF4 to its target gene COMPASS‐like.	[22]
REF6‐PIF4	Promotes the binding of PIF4 to its target gene.	[23]
GID1‐SCFSLY1/GID2	Promotes expression of flowering genes	[35, 36, 37]
COP1‐DELLA	Promotes the ubiquitination and degradation of DELLA proteins	[38]
MED15‐DELLA	Promotes DELLA degradation	[39]
PIF4‐BES1	Represses *DWF4* expression	[41, 42]
PhyB‐PIF4	Promotes PIF4 degradation	[47]
CRY1‐TCP17	Represses *PIF4* expression	[50]
UVR8‐PRC2	Promotes *miR156* expression	[54]
UVR8‐HFR1	Represses *PIF4* expression	[54]
PhyB/CRY1/UVR8‐COP1	Promotes the release of HY5	[136, 137, 138, 139]
ELF3‐ELF4‐LUX	Represses *PIF4* and *PIF5* expression	[57]
XBAT31‐BBX18‐ELF3	Promotes ELF3 degradation	[59]
SHB1‐CCA1‐LHY	Promotes *PIF4* expression	[73]
GmCRY1s‐DELLA	Enhance the stability of DELLA proteins	[77]
ZmNF‐YA3‐ZmCO‐like‐ZmFPF1	Promotes *ZmFT‐like12* expression	[69]
CLV3‐CLV1‐CLV2	Represses *WUS* expression	[82]
CLE40‐BAM1	Promotes *WUS* expression	[85]
CLV3‐RPK2	Represses *WUS* expression	[86]
CIK‐CLV1‐CRN‐RPK2	Transmits CLV3 signals	[87]
WUS‐LFY	Promotes *AG* expression	[88]
AG‐PRC2	Represses *WUS* expression	[140]
AG‐KNU‐PRC2	Represses *WUS* expression	[89]
EPFLs‐ERf	Promotes *MPK3/6* expression	[94, 95]
MPK3/6‐SPL	Promotes the phosphorylation of SPL	[94, 95]
MPK3/6‐WRKY34/WRKY2	Promotes *GPT1* expression	[96]
EMS1‐SERK1	Recognizes TPD1	[97, 98]
EMS1‐TPD1	Promotes *BES1* expression	[100]
CLE19‐PXL1	Promotes the phosphorylation of PXL1	[106]
EPFL‐ERf‐SERK	Regulates the development of the outer integument	[109]
CLE40‐ACR4‐CIK	Represses *WOX5* expression	[110]
RALF4/19‐BUPS‐ANX	BUPS‐ANX recognizes and binds the autocrine peptide RALF4/19	[141]
RALF34‐BUPS‐ANX	RALF34 competes for interaction with BUPS‐ANX	[121]
RALFs‐FER‐LRE	Promotes Ca^2+^ entry into synergid cells	[122]

Although BR signaling has been clearly implicated in thermoresponsive flowering, current knowledge largely centers on its interplay with PIF4. Whether BRs modulate other key flowering regulators under elevated temperatures remains unknown. This knowledge gap extends to additional temperature‐responsive hormones. For example, elevated temperatures induce the accumulation of ethylene, abscisic acid (ABA), and salicylic acid (SA) [43, 44], yet their mechanistic roles in thermosensitive flowering remain poorly resolved. Elucidating how plants integrate these multifaceted hormonal cues to fine‐tune flowering transitions under heat stress represents a critical frontier in developmental biology and a prerequisite for breeding crops with enhanced resilience to climate change.

### 2.5 Photoreceptors

Recent findings highlight plant photoreceptors as central players in temperature perception. Among them, phytochrome B (phyB) acts as a dual light–temperature sensor, interconverting between two photoreversible isoforms: the red light–absorbing Pr (inactive) form and the far‐red light–absorbing Pfr (active) form. These conformers interconvert in response to both light and temperature cues [45]. Under normal temperature conditions, phyB suppresses flowering through multiple regulatory pathways. Specifically, in high red:far‐red (R/FR) environments, Pfr–phyB represses *CONSTANS (CO)*, reducing *FT* expression and delaying flowering [46]. In addition, Pfr–phyB functions as a potent negative regulator of thermomorphogenesis by directly binding *PIF4* and targeting it for ubiquitin‐dependent proteasomal degradation [47]. However, elevated temperatures shift phyB from its bioactive Pfr state to the inactive Pr form, disrupting phyB–*PIF4* interactions. As a result, *PIF4* stabilizes and activates flowering gene transcription. This mechanism operates alongside other photoreceptors, including CRY1 and UVR8, which also undergo temperature‐dependent inactivation to mediate thermosensing (Figure 1A,B and Table 1).

Cryptochromes (CRYs), a family of conserved blue‐light photoreceptors, also display thermosensory activity. Recent studies demonstrate that CRYs undergo accelerated inactivation under combined dark and high‐temperature conditions [48], implicating them in thermal perception. In particular, CRY1 exerts precise control over thermomorphogenesis by modulating *PIF4* activity in a temperature‐dependent manner [49]. Mechanistically, at lower temperatures, CRY1 forms a stable complex with TCP17, sequestering it from the *PIF4* promoter. Elevated temperatures disrupt the CRY1–TCP17 interaction, releasing TCP17 to activate *PIF4* transcription [50]. This CRY–TCP17–*PIF4* module thus functions as a temperature‐sensitive molecular switch, conferring plasticity to flowering responses under fluctuating conditions.

Another photoreceptor, UVR8, integrates both UV‐B and thermal cues [51, 52]. Acting as a negative regulator of flowering, UVR8 represses floral induction through two interconnected pathways. Under UV‐B exposure, monomeric UVR8 suppresses Polycomb Repressive Complex 2 (PRC2) activity, reducing H3K27me3 deposition at the miR156 locus and thereby activating this flowering repressor [53]. In parallel, UVR8 stabilizes the bHLH transcription factor HFR1, which physically interacts with and inhibits *PIF4* [54]. This dual repression is dynamically modulated: darkness and elevated temperatures promote RUP‐mediated UVR8 dimerization, inactivating the receptor and relieving flowering repression. Thus, UVR8 functions as a key integrator that converts both light and temperature inputs into developmental responses via its dynamic monomer–dimer transitions.

Although substantial evidence demonstrates that phyB, CRY1, and UVR8 undergo temperature‐dependent inactivation to regulate thermosensing [45, 48, 51], the downstream regulatory networks orchestrating high‐temperature responses remain only partially resolved. Deciphering these architectures will be essential for constructing a comprehensive model of how plants integrate environmental cues to precisely calibrate flowering time under thermal stress.

### 2.6 Liquid–Liquid Phase Separation (LLPS)

Beyond well‐established transcriptional and post‐translational mechanisms, recent discoveries highlight liquid–liquid phase separation (LLPS) as a novel regulatory layer in temperature‐mediated flowering, potentially representing an evolutionary strategy for coping with environmental fluctuations. LLPS is a physicochemical process in which biomolecules condense into membraneless compartments to spatially organize cellular reactions [55, 56]. A prime example is ELF3, a central thermosensory protein that controls flowering via two distinct mechanisms. First, ELF3 associates with ELF4 and the MYB transcription factor LUX ARRHYTHMO (LUX) to assemble the Evening Complex (EC; ELF3–ELF4–LUX), which represses PIF4/5 transcription and delays flowering [57]. Elevated temperatures destabilize this complex by promoting XBAT31‐mediated degradation of ELF3, thereby relieving repression of flowering genes [58, 59]. Second, ELF3 functions as a direct thermosensor through its prion‐like domain (PrD), which undergoes phase separation in response to temperature fluctuations [60]. At 22 °C, ELF3 remains diffusely distributed in the nucleus, binds DNA, and represses target gene expression. By contrast, elevated temperatures induce condensate formation, abolishing its DNA‐binding ability and accelerating flowering [61]. Emerging evidence further suggests that ELF3 does not act in isolation, but instead serves as a central transcriptional hub through extensive interactions with other transcription factors [62, 63]. Thus, future work should focus on identifying ELF3‐interacting partners and determining how LLPS modulates these protein–protein interactions in response to temperature, which will be critical for elucidating the mechanistic basis of temperature‐dependent flowering [62, 63, 64].

Photoreceptors also employ LLPS to mediate temperature sensing. Under ambient light, phyB translocates from the cytoplasm into the nucleus, where it forms subnuclear condensates termed photobody nuclear bodies (PBs) [65]. These PBs sequester and inhibit PIF4 activity [66]. High temperatures are detected specifically by the N‐terminal extension (NTE) domain of phyB, which triggers PB dissociation and releases *PIF4*, thereby activating transcription of flowering‐related genes [66]. Despite these insights, the current catalog of LLPS‐associated thermosensory proteins remains limited. Expanding this repertoire and mechanistically dissecting their roles will not only deepen our understanding of plant adaptation to fluctuating environments but also provide promising molecular targets for breeding strategies aimed at stabilizing yield and promoting early maturation under climate stress.

## 3 Thermally Regulated Flowering Mechanisms in Major Crops

The theoretical framework of temperature‐mediated flowering has been extensively validated in major crops such as soybean, rice, maize, and tomato, highlighting both the evolutionary conservation of thermosensory mechanisms and their practical importance for crop breeding [67, 68, 69, 70]. In soybeans, elevated temperatures strongly influence flowering by modulating the expression of core photoperiod‐pathway genes through a dual regulatory strategy. High temperatures suppress the flowering repressors *E1* and *E2* family genes, thereby alleviating their inhibitory effects, while simultaneously activating flowering promoters including *GmFT2a*, *GmFT5a*, *GmCOL5a/5b*, and *GmCOL6a/6b*, collectively accelerating the floral transition. In addition, microRNA‐mediated post‐transcriptional control plays a pivotal role: high temperatures reduce *miR156* abundance, relieving repression of its target *SPL* genes and promoting flowering [67] (Figure 1C).

Flowering time in rice (*Oryza sativa* L.) is governed by an intricate genetic network that integrates environmental and developmental signals. Central to this system is *Ehd1* (*Early Heading Date 1*), which activates the florigen genes *Hd3a* and *RFT1* (Chen et al., 2018). These florigens are transported from leaves to the shoot apical meristem, where they initiate floral transition, making their regulation critical for reproductive timing [68]. Recent findings reveal that rice finely adjusts flowering in response to temperature fluctuations through dynamic regulation of flowering repressors and promoters. A key regulator is OsGhd7, which exhibits temperature‐sensitive expression. Under cool conditions, elevated OsGhd7 levels strongly repress the Ehd1–RFT1 pathway, delaying flowering as a potential adaptive strategy against unfavorable environments [71]. By contrast, warm temperatures markedly reduce *OsGhd7* expression, releasing repression of *RFT1* and accelerating flowering. Beyond the *OsGhd7–Ehd1* module, *OsPRR37* has emerged as an additional thermoresponsive regulator, likely acting through circadian modulation or direct regulation of flowering genes [72]. These findings underscore the sophistication of rice's multilayered thermal adaptation system. Future studies should aim to identify the primary thermosensors and dissect the crosstalk among these regulatory modules to fully elucidate how rice integrates diverse environmental signals for optimal flowering time control (Figure 1C).

In maize, preliminary studies suggest that the nuclear factor‐Y (NF‐Y) complex plays a central role in regulating thermal responses during flowering. At the molecular level, *ZmNF‐YA3* interacts with *ZmCO‐like* and *ZmFPF1* to form a transcriptional complex that directly activates *ZmFT‐like12* expression by binding its promoter, thereby initiating flowering. Furthermore, *ZmNF‐YA3* specifically binds the promoters of heat‐responsive genes such as *ZmbHLH92*, *ZmFAMA*, and *ZmMYC4*, linking thermosensory flowering with heat‐stress adaptation [69]. Despite these advances, the mechanisms underlying temperature‐mediated flowering in other major crops, including cotton, remain largely unresolved. The genomic complexity and genetic diversity of many crops, coupled with the absence of systematic molecular frameworks, continue to impede the application of precision breeding for climate resilience. Future research should prioritize examining flowering under dynamic temperature regimes and integrating artificial intelligence and big‐data approaches to accurately predict flowering time across variable environments, thereby supporting the development of temperature‐resilient cultivars.

## 4 Molecular Integrations of Photothermal Signals in Flowering Control

Light and temperature act as dominant environmental cues regulating flowering, with their signaling pathways exhibiting extensive crosstalk. In natural settings, intense light or extended photoperiods often coincide with elevated temperatures, suggesting that photoreceptors and downstream effectors coordinate both photomorphogenic and thermosensory responses. Dissecting the molecular mechanisms of this integration is vital for breeding climate‐resilient crops and securing agricultural productivity under global warming.

**FIGURE 2:**
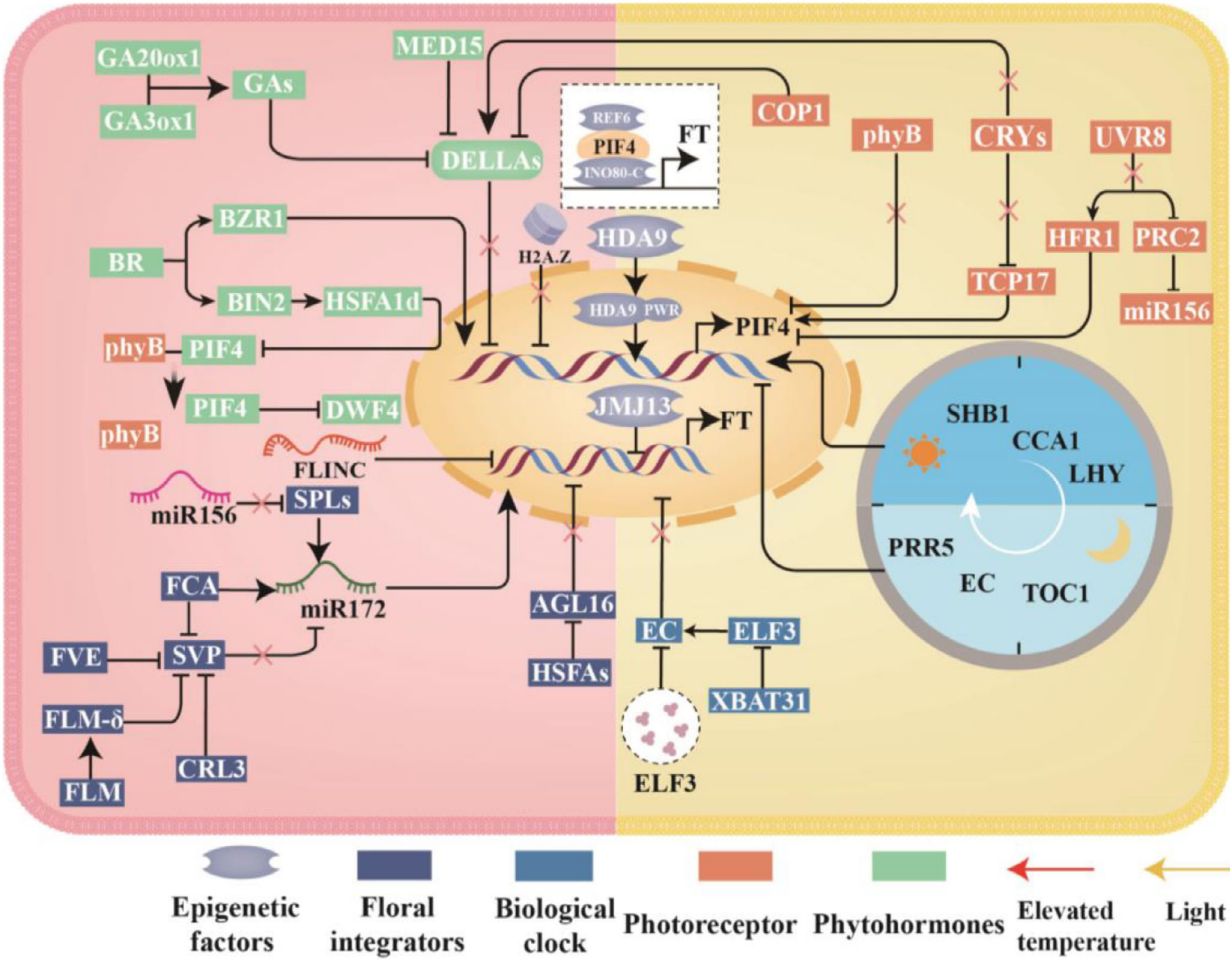
Integration of light and temperature signaling in the regulation of flowering. FT acts as a central signaling hub, integrating light cues (circadian clock, photoperiod, and light signaling transduction) and temperature inputs (phytohormones, epigenetics, and thermosensors) to mediate a multidimensional regulatory network for flowering. The red region on the left highlights signaling pathways responsive to high temperatures, while the yellow region on the right depicts the light regulatory network under high‐temperature conditions.

The bHLH transcription factor PIF4 functions as a master regulator of this integration. PIF4 displays robust diurnal oscillations, peaking at midday and declining at night, controlled by circadian regulators including CCA1, LHY, and the EC complex [73]. In the morning, SHORT HYPOCOTYL UNDER BLUE1 (SHB1) interacts with CCA1 and LHY to recruit them to the PIF4 promoter, enhancing transcription [73]. In contrast, evening accumulation of the EC complex represses PIF4 by binding LBS cis‐elements in its promoter [74]. At night, PRR family members PRR5 and TOC1 further suppress *PIF4* transcription, collectively generating its rhythmic pattern. Beyond this circadian regulation [74, 75], PIF4 also serves as a key thermosensor, with its stability and transcriptional activity dynamically modulated by interactions with phyB, HMR, PIF7, and HFR1. This dual responsiveness positions PIF4 as a molecular integrator that translates concurrent light and temperature signals into flowering decisions, ensuring optimal reproductive timing under variable environments (Figure 2).

The GA pathway provides an additional layer of photothermal integration. Elevated temperatures induce *GA20ox1* and *GA3ox1*, leading to the accumulation of bioactive GA4 and activation of GA signaling. In parallel, phyB‐mediated light signaling enhances *AtGA20ox2* expression, further stimulating GA biosynthesis. Elevated GA levels then activate flowering regulators, including *FT*, *TSF*, and *SP* [76]. Recent findings in soybean and *Arabidopsis* demonstrate that blue light–activated cryptochromes (*GmCRY1s*/*AtCRYs*) physically interact with and stabilize DELLA proteins [77], a mechanism also validated in *Arabidopsis* [78]. This establishes a direct molecular link between light perception and GA signaling, highlighting GA as a central integrator of environmental cues in the regulation of flowering transitions.

## 5 Regulation of Flower Development by Small Peptides and Receptor‐Like Kinases

**FIGURE 3:**
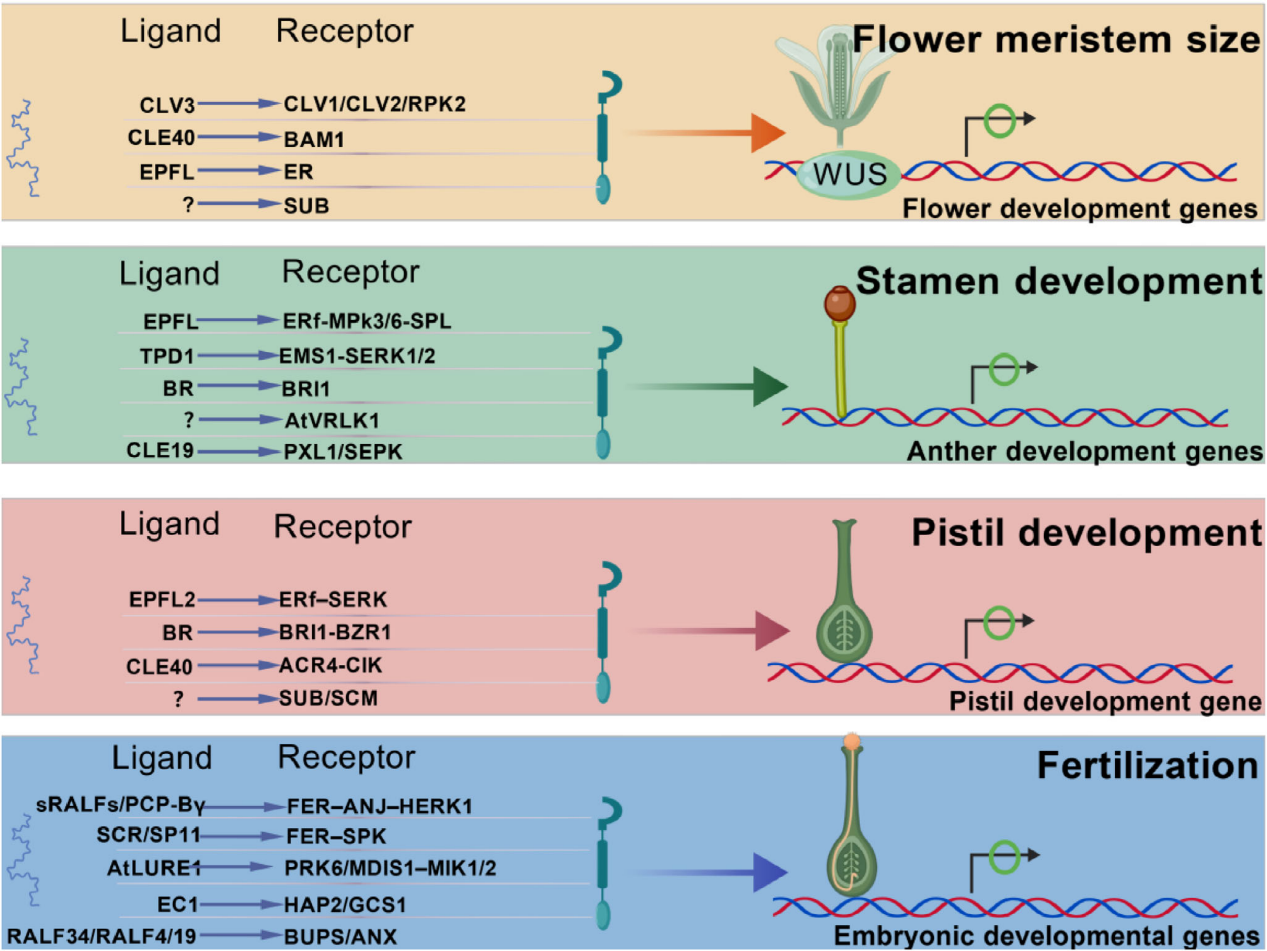
Receptor‐like kinases in floral development. This diagram illustrates the diverse roles of RLKs and their ligands in flower development, categorized into four major functional groups: regulation of floral meristem size, stamen morphogenesis, pistil differentiation, and fertilization.

Environmental and endogenous cues converge on the FT protein, which is transported from leaves to the floral meristem, where it activates key pathways driving floral organ initiation and reproductive development. Increasing evidence indicates that small peptides, functioning as signaling molecules, play essential roles in regulating floral meristem activity, stamen differentiation, carpel morphogenesis, and fertilization in a spatiotemporally precise manner. These peptides exert their effects by binding to receptor‐like kinases (RLKs), which initiate downstream signaling cascades [79] (Figure 3, Table 3).

**TABLE 3:** Functional roles of flower development factors in plants.

*WUS* (*WUSCHEL*)	Coordination *LFY*, activate *AG* expression	Ensure the continuous growth of the meristem	[81, 88]
*LFY*	Coordination *WUS*, activate *AG* expression	Ensure the continuous growth of the meristem	[88]
*AG*	Represses *WUS* expression, encodes *KNU* expression	The activity of the flower meristem is terminated	[89, 140]
*KNU* (*KNUCKLES*)	Combination with *PRC2* represses *WUS* expression	The activity of the flower meristem is terminated	[89]
*SPL*/*NZZ* (*SPOROCYTELESS*)	Interacts with *MPK3*/*6*	Initiation of the archesporial cells in the adaxial anther lobes	[94, 95]
*MPK3*/*MPK6*	Phosphorylate *WRKY34* and *WRKY2*	Control lipid body accumulation during pollen maturation	[96]
*WRKY34*,*WRKY2*	unknown	Control lipid body accumulation during pollen maturation	[96]
*GPT1*	Transport *Glc6P*	Control lipid body accumulation during pollen maturation	[96]
*AtVRLK1*	unknown	Affects anther rupture and regulates pollen development	[102]
*INO*	unknown	The number of embryo beads and seeds increased	[111, 112]
*WOX5 (WUSCHEL RELATED HOMEOBOX 5)*	unknown	Maintain the columella stem cells in the root	[110]
*AtEC1.1*‐*AtEC1.5*	*EC1* homologous gene	Specifically expressed in female reproductive tissues	[124]

### 5.1 Development of the Floral Meristem

The precise regulation of floral meristem size is fundamental to reproductive success, requiring tight coordination among multiple signaling pathways. Central to this process is the CLV–WUS negative feedback loop, which maintains meristem homeostasis by balancing stem cell proliferation and differentiation [80]. The transcription factor *WUSCHEL* (*WUS*), produced in the organizing center, diffuses into adjacent stem cell domains to sustain their activity and promote proliferation, thereby supporting meristem growth [81]. In contrast, *CLAVATA3* (*CLV3*), a secreted CLE‐family peptide, restricts WUS activity by binding to the receptor complex CLV1/CLV2. This repression curtails excessive proliferation and restores stem cell division to a steady state [82]. Thus, the opposing actions of WUS‐driven proliferation and CLV3‐mediated inhibition establish a robust feedback circuit that ensures stable control of meristem size [80].

This canonical pathway is reinforced by additional, spatially distinct mechanisms. BARELY ANY MERISTEM 1/2/3 (BAM1/2/3), homologs of CLV1, contribute to the regional regulation of meristem activity. Whereas the CLV1/CLV3 complex represses *WUS* in the central zone of the shoot apical meristem (SAM), BAM1/CLE40 signaling promotes *WUS* expression in the peripheral zone, generating a balanced regulatory network [83, 84, 85]. The receptor‐like kinase *RECEPTOR‐LIKE PROTEIN KINASE 2 (RPK2)*, also called *TOADSTOOL 2 (TOAD2)*, provides an additional regulatory input, as *rpk2* mutants display stem cell overproliferation phenotypes similar to *clv* mutants [86]. Recent advances have identified *CLAVATA3 INSENSITIVE KINASEs (CIKs)* as coreceptors that associate with *CLV1*, *CRN*, and *RPK2*. Upon phosphorylation, CIKs mediate *CLV3* signaling and repress meristem expansion, revealing a multilayered receptor architecture that underpins meristem maintenance [87].

**FIGURE 4:**
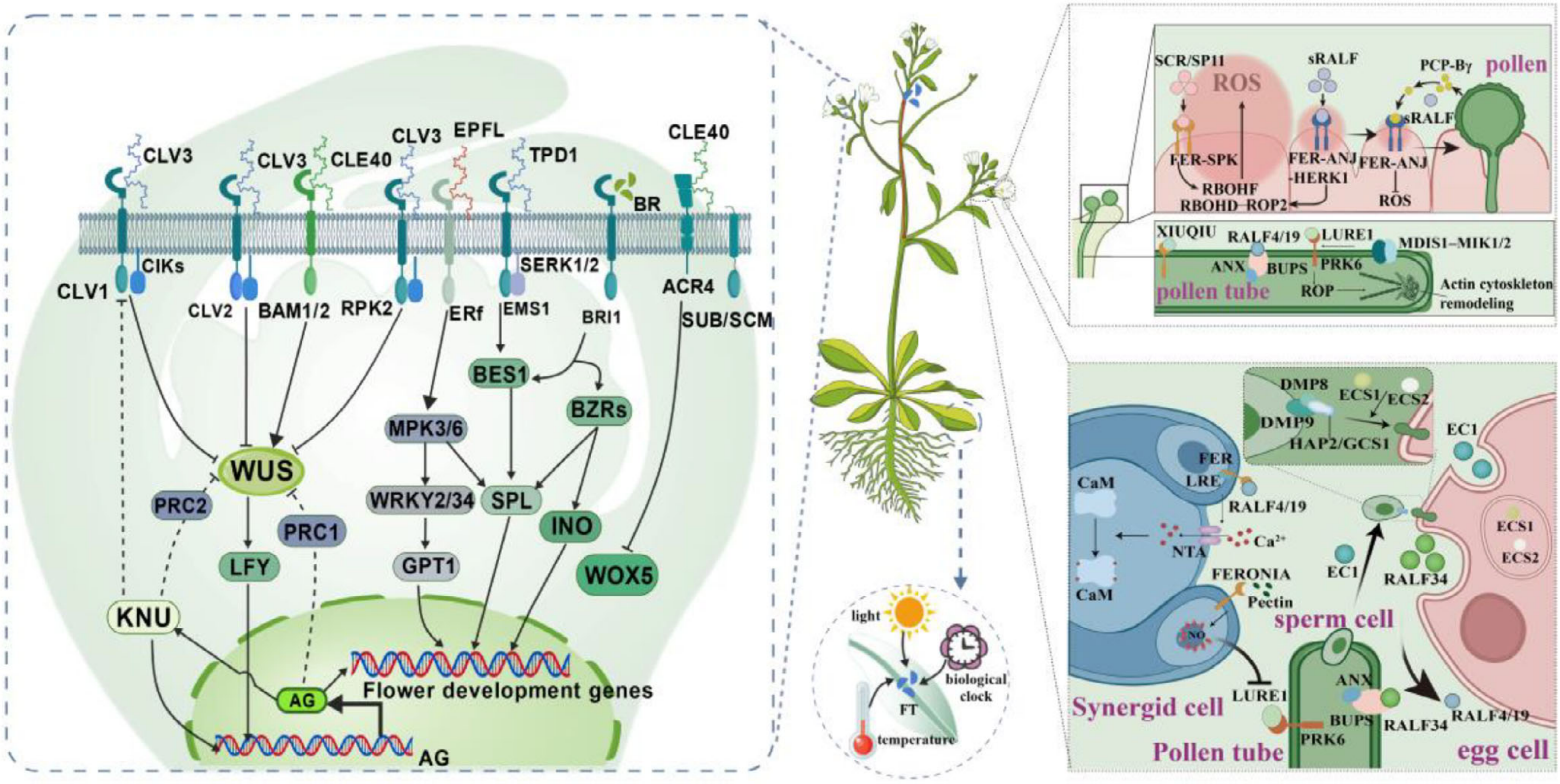
Receptor‐like kinase‐mediated signalling pathways in reproductive development. During floral induction, various environmental and endogenous signals converge to regulate the florigen (FT) protein, which is transported from leaves to the shoot apical meristem (SAM) to initiate flowering. Within the SAM, a complex network of receptor kinases and small peptides orchestrates floral development with high spatiotemporal precision. These regulatory mechanisms govern critical processes, including floral meristem maintenance, organ development, and gamete fusion. The left panel illustrates key floral organ development pathways: the CLV3‐CLV1/2‐WUS module maintains meristem homeostasis; the EPFL‐ERf and TPD1‐EMS1‐SEK1/2 modules regulate pollen development; and the BR‐BRI1 and CLE40‐ACR4 modules control ovule development. The right panel details the double fertilization process, encompassing stigma–pollen recognition, pollen tube growth, and the fusion of male and female gametes.

Termination of meristem activity involves another feedback system centered on WUS and AGAMOUS (AG). Together with LEAFY (LFY), WUS activates AG expression [88]. Accumulated AG then enforces meristem termination by two parallel mechanisms: (i) recruiting Polycomb Repressive Complex 1 (PRC1) to silence WUS, and (ii) inducing KNUCKLES (KNU), a C2H2‐type zinc‐finger transcription factor that recruits PRC2 to further repress WUS [89]. These AG–PRC1 and AG–KNU–PRC2 feedback loops not only terminate stem cell activity but also activate carpel differentiation genes, ensuring a smooth transition to reproductive development. Beyond CLV‐related signaling, additional receptor‐like kinases (RLKs) independently contribute to meristem regulation. The ERECTA family (ER, ERL1, ERL2) and the LRR‐RLK SUB modulate floral meristem and organ development independently of CLV signaling [90, 91]. Collectively, these multiple parallel systems highlight the evolutionary robustness of meristem regulation (Figure 4).

### 5.2 Pollen Development

Pollen development in angiosperms is coordinated by an elaborate network of signaling pathways that regulate the sequential stages from anther initiation to pollen maturation. A key regulatory module involves cysteine‐rich receptor‐like kinases (CIKs), which act as coreceptors for BAM1/2 and RPK2. Their phosphorylation is essential for proper archesporial cell division and parietal layer formation; mutations cause severe defects, including loss of parietal layers and excessive proliferation of microspore mother cells [92, 93]. In parallel, the ERECTA family (ERf), together with its ligand EPFL, forms a receptor complex that activates MPK3/6. These kinases regulate pollen development through dual mechanisms: they phosphorylate SPOROCYTELESS (SPL) to trigger archesporial cell initiation in the adaxial anther lobes [94, 95], and they phosphorylate WRKY34 and WRKY2, which activate *GPT1* expression to promote glucose‐6‐phosphate (Glc6P) transport into plastids, thereby supporting lipid accumulation crucial for pollen maturation [96]. These findings illustrate how plants integrate transcriptional and metabolic pathways to ensure precise developmental transitions during pollen formation (Figure 4 and Table 3).

A second critical module is the TPD1–EMS1–SERK1/2 cascade. Here, the cysteine‐rich peptide TPD1 signals through the receptor kinase EMS1 and its coreceptors SERK1/2 to coordinate tapetum specification and microspore mother cell development [97, 98]. This pathway also interfaces with BR signaling: gain‐of‐function mutants (*bzr1‐1D*, *bes1‐D*) partially rescue the defective anther phenotypes of *ems1*, *tpd1*, and *serk1serk2* mutants [99, 100]. Mechanistically, the EMS1–TPD1 complex promotes BES1 dephosphorylation, activating it to regulate anther development genes such as *SPL*/*NZZ*. Thus, BES1 functions as a signaling hub, activated both via the canonical BRI1–BR–BAK1 pathway and independently through EMS1–TPD1–SERK1/2, enabling plants to coordinate reproductive development under diverse internal and environmental contexts [100]. Consistent with this, mutations in BRI1 (bri1‐116) result in severe reproductive defects, including tapetal disorganization, failed pollen release, and reduced fertility [101].

Beyond these well‐characterized pathways, emerging studies identify additional RLKs that fine‐tune pollen development. In Arabidopsis, AtVRLK1 regulates anther dehiscence [102], while in rice, the LRR II‐RLKs TMS10 and TMS10L ensure timely tapetum degeneration and pollen viability [103]. The CRRLK proteins DRUS1 and DRUS2 suppress premature cell death and modulate sugar metabolism during anther development [104], whereas OsLecRK5 promotes callose biosynthesis in meiosis via phosphorylation of UGP1 [105]. Moreover, the CLE19–PXL1/SERK module exemplifies peptide–receptor regulation of tapetum and pollen formation [106]. Collectively, these pathways highlight the remarkable complexity and redundancy of the signaling networks governing pollen development, in which multiple layers of regulation ensure the precise coordination of anther lobe initiation, sporocyte division, tapetum specification, and pollen maturation. Such sophisticated regulatory architecture not only secures reproductive success but also provides adaptive flexibility to environmental and developmental signals.

### 5.3 Carpel Development

Carpel development, a central determinant of reproductive success in flowering plants, is governed by a complex interplay of receptor‐like kinase (RLK) signaling networks and hormonal pathways. Recent studies highlight strong functional parallels between stamen and carpel development, particularly via ERf‐family and BRI1‐mediated signaling cascades [108, 109, 110].

The ERf‐family plays a pivotal role in ovule morphogenesis, regulating processes from ovule primordium initiation to outer integument elongation and embryo sac development. Within this pathway, the secreted peptide EPFL2 functions as a key ligand, binding to ERL1 and ERL2 to initiate ovule primordium formation [107]. EPFL2 also activates the MPK3/6 cascade, driving outer integument cell division and influencing ovule development [108]. SERK co‐receptors are indispensable in this pathway, as *serk1*/*2*/*3* triple mutants exhibit severe defects in integument elongation and embryo sac formation [109]. Collectively, the EPFL2–ERf–SERK module emerges as a master regulator of ovule and integument development in *Arabidopsis*.

Additional RLK pathways further refine ovulogenesis. *ABNORMAL LEAF SHAPE 2 (ALE2)* regulates epidermal cuticle biosynthesis, with *ale2* mutants showing pleiotropic integumental and embryo sac defects. The RLK *SUB/SCM* exerts more specialized control over outer integument morphogenesis, where loss‐of‐function mutants display characteristic scoop‐like malformations and embryo sac abortion [91]. The non‐LRR RLK *ACR4* is likewise essential for integument development; *acr4* mutants show irregular outer integument growth and developmental delay. Importantly, the ACR4–CIK receptor complex perceives CLE40 signaling to suppress *WOX5* expression, maintaining columella stem cell stability [110].

Hormonal regulation, particularly via BR signaling, also plays a critical role. Mutants in the BR receptor *BRI1* (*bri1‐116* and *bri1‐701 brl1/3*) exhibit incomplete outer integument formation, leaving the inner integument and nucellus partially uncovered [111]. The BR transducer BZR1 enhances ovule development by activating target genes such as *INO*; its gain‐of‐function allele *bzr1‐1D* produces increased ovule and seed numbers [111, 112]. Conversely, the BR biosynthesis mutant *det2* reduces ovule and seed set, whereas loss of the negative regulator *BIN2* markedly increases them [113]. Together, these findings underscore how BR signaling integrates with RLK modules to establish a robust, multilayered network that ensures the precise spatiotemporal regulation of carpel formation.

### 5.4 Fusion of Male and Female Gametophytes

The coordinated recognition and fusion of male and female gametophytes constitute the core of sexual reproduction in angiosperms, governing critical processes such as pollen–stigma interaction, pollen tube guidance, sperm release, gamete fusion, and polyspermy prevention. These events are mediated by specific peptide–receptor interactions, which provide the molecular precision required for reproductive success.

The first barrier is established at the stigma surface, where papilla cells regulate pollen acceptance through a reactive oxygen species (ROS)‐dependent mechanism. ROS production, primarily mediated by NADPH oxidases (*e.g*., RBOHD, RBOHF), is controlled by CrRLK1L‐family receptors (FER, ANJ, HERK1) and stigma‐derived peptides. In *Arabidopsis*, sRALF1/22/23/33 peptides bind the FER–ANJ–HERK1 complex, activating the ROP2–RBOHD pathway to sustain high ROS levels and reject heterospecific pollen [114]. In self‐incompatible plants such as *Brassica rapa*, the SRK receptor recognizes self‐pollen SCR/SP11, forming a complex with FER to activate RBOHF‐dependent ROS production, thereby blocking self or distant pollen [115]. Compatible pollen circumvents this barrier by secreting competing peptides, such as PCP‐Bγ, which bind FER–ANJ more strongly than sRALFs, suppressing ROS and enabling pollen hydration and germination [116].

During pollen tube growth, autocrine and paracrine signaling ensure directional guidance and controlled sperm delivery. Autocrine peptides RALF4/19 maintain tube integrity via the BUPS–ANX receptor complex, preventing premature sperm release [117]. Female‐derived attractants provide guidance: synergid‐secreted AtLURE1 peptides bind PRK6 on pollen tubes, activating ROP signaling and directing actin remodeling at the tube apex [118]. The MDIS1–MIK1/2 receptor complex enhances sensitivity to LURE1, and mutants in *mdis1* or *mik1 mik2* exhibit impaired guidance and reduced fertilization [119]. Synergid‐secreted XIUQIU1–4 peptides act as conserved attractants for closely related species via a PRK6‐independent pathway, ensuring robust fertilization alongside AtLURE1 [120].

Upon reaching the ovule, the pollen tube undergoes a tightly regulated rupture to release sperm cells, a decisive step controlled by complex signaling networks. As fertilization approaches, the ovule‐derived ligand RALF34 competes with the pollen tube's autocrine peptides RALF4/19 for binding to the BUPS–ANX receptor complex. This competitive interaction functions as a molecular switch that induces pollen tube rupture, ensuring timely sperm release and fertilization success [121]. Redundant regulation is provided by the FER–LRE–NTA cascade: when pollen tube‐derived RALFs are perceived by FER on synergid cells, FER–LRE recruits the Ca^2^^+^ channel protein NTA to the plasma membrane, where it assembles an active Ca^2^^+^ influx channel. This Ca^2^^+^ elevation in the synergid triggers pollen tube rupture and sperm release [122]. Beyond this, FERONIA coordinates pollen tube reception via a pectin–nitric oxide (NO) signaling module. By modulating the de‐esterification of pectin in the filiform apparatus—a specialized synergid structure—FER establishes the physical and biochemical foundation for downstream signaling. Upon pollen tube arrival, pectin fragments activate FER‐dependent pathways that induce NO accumulation. Elevated NO then terminates the attraction signal through two mechanisms: (i) S‐nitrosylation of the LURE1 precursor prevents its secretion, and (ii) inhibition of mature LURE1 binding to its receptor PRK6 on the pollen tube. Together, these processes ensure precise temporal control of fertilization [123].

Although not yet fully resolved, the molecular basis of gamete fusion is increasingly understood. EC1 peptides—cysteine‐rich proteins secreted by the egg cell—are key regulators. In *Arabidopsis*, five homologs (AtEC1.1–AtEC1.5) are specifically expressed in female reproductive tissues [124]. Upon sperm arrival, vesicles release EC1 peptides via exocytosis, which induce the fusogen HAPLESS2/GENERATIVE CELL SPECIFIC1 (HAP2/GCS1) to translocate from internal vesicles to the sperm surface, priming it for fusion [125, 126]. Subsequent membrane merger is facilitated by DMP8 and DMP9, likely by modulating membrane curvature [127]. Additionally, egg‐secreted aspartic proteases ECS1 and ECS2 reinforce sperm–egg adhesion, ensuring preferential fertilization of the egg cell [128].

Polyspermy is prevented by a dual safeguard system. The fertilized egg secretes ECS1/2 peptides that cleave the conserved C‐terminal domain of LURE1, thereby inactivating this chemoattractant. Simultaneously, the first penetrating pollen tube releases RALF6/7/16/36/37 peptides, which bind to the FER–ANJ–HERK1 receptor complex on septum cells and activate a signaling cascade that blocks further pollen tube entry [129, 130]. If fertilization fails, however, the unfertilized central cell secretes chemoattractant peptides SAL1/2 to recruit additional pollen tubes, providing a fail‐safe mechanism [131].

In summary, male–female gametophyte interactions are orchestrated through a multilayered signaling network, in which small peptides (LURE1, RALF, PCP‐B) and receptor kinases (FER, PRK6, MDIS1) form the core regulatory modules. This system ensures species‐preferential fertilization, prevents polyspermy, and enables recovery mechanisms in the event of fertilization failure, while also safeguarding reproductive isolation. These insights not only deepen our understanding of plant reproductive biology but also identify molecular targets of high potential for crop improvement and hybrid breeding strategies.

## 6 Discussion

As sessile organisms, plants have evolved highly sophisticated adaptive strategies through long‐term natural selection and domestication, allowing them to survive and reproduce under dynamic fluctuations in light and temperature. These strategies rely on precise environmental sensing systems that perceive external cues and translate them into developmental responses through complex regulatory networks. Such evolutionary innovations have enabled plants not only to optimize flowering time for reproductive success but also to expand their cultivation range across diverse climatic regions.

Molecular studies have revealed that the regulation of flowering and reproduction depends on intricate spatiotemporal coordination of multiple genetic and physiological factors. This knowledge has provided critical guidance for crop improvement, particularly in addressing challenges posed by climate variability and geographical expansion [132]. For example, molecular breeding programs that exploit early‐flowering alleles have successfully shortened the growth cycle of crops such as cotton, maize, and soybean, mitigating yield losses caused by seasonal fluctuations. Manipulation of floral regulators, such as MADS58 in rice, has also demonstrated potential to improve yield by inducing carpel duplication. Beyond yield enhancement, flowering‐time genetics has been pivotal in expanding the latitudinal range of cultivation. Traditional photoperiod‐sensitive varieties often experience flowering delays at higher latitudes, leading to severe yield reductions. Strategic selection of early‐flowering genotypes in rice and maize has circumvented daylength constraints, enabling stable production across wider geographic ranges [133]. These examples underscore how fundamental discoveries in reproductive physiology can be rapidly translated into agricultural solutions.

Despite significant progress, important gaps remain in our understanding of how plants adapt reproductive processes to fluctuating environments. First, recent findings show that certain plant proteins undergo liquid–liquid phase separation (LLPS) in response to light and temperature fluctuations, thereby regulating growth, development, and reproduction [134, 135]. This mechanism represents an efficient strategy for rapid adaptation to unpredictable environmental cues. While some flowering regulators have been implicated in LLPS‐mediated control of flowering, such mechanisms remain largely unexplored in crops. Targeted protein engineering to modulate phase‐separation properties of flowering regulators may offer a powerful alternative to conventional genetic approaches for optimizing flowering traits.

A second major challenge lies in resolving trade‐offs between early maturity and other essential agronomic traits. Although flowering‐time genes have been effectively used to generate early‐maturing cultivars, these improvements often compromise yield potential or stress resilience [132]. Future research must prioritize the identification of superior alleles and innovative regulatory strategies that simultaneously confer early maturity, stress tolerance, and high yield. Third, our current knowledge of reproductive physiology remains fragmented across developmental stages and tissues, leaving the spatiotemporal dynamics of regulatory mechanisms insufficiently understood. Recent studies highlight coordination between shoots and roots in heat responses: for instance, the HY5/PIF module in shoots regulates auxin biosynthesis, which in turn directs thermomorphogenic responses in roots. These findings emphasize the need to dissect organ‐specific roles in environmental sensing and reproductive regulation. Cutting‐edge single‐cell and spatial transcriptomic approaches provide unprecedented opportunities to unravel these dynamics at high resolution, offering insights into how plants integrate environmental signals across tissues and developmental stages to secure reproductive success. Finally, research on the molecular mechanisms underlying plant reproductive physiology is largely conducted under controlled conditions, which fail to reflect the complexity of fluctuating natural environments. Integrating theoretical research with field experiments is therefore crucial for a comprehensive understanding of gene functions under natural conditions and for accelerating the translation of basic research into agricultural applications. By addressing these gaps, future research can establish the mechanistic foundation for designing next‐generation crops with enhanced adaptability to environmental fluctuations and improved yield stability.

## Author Contributions

X.Y.G., F.G.L., and H.Z. designed this study; S.H.H., Y.X., S.X.Z., L.G.L., Y.H.L., and C.Y.J. revised the manuscript and prepared the tables; all authors approved the final version of the manuscript.

## Funding

This work was supported by the Natural Science Foundation of Shandong Province (ZR2022QC003), the National Natural Science Foundation of China (32201762, 32522074), Innovation Program of Chinese Academy of Agricultural Sciences (CAASCSIAF‐202402), China Agriculture Research System of MOF and MARA (CARS‐15‐02), and the China Postdoctoral Special Funding Project (2025T181104).

## Conflicts of Interest

The authors declare no conflicts of interest.

## Data Availability

The authors have nothing to report.
